# Supplementary material for: Validation of the English Version of the General Dietary Behavior Inventory (GDBI-E)
Source: Int J Environ Res Public Health. 2022 Mar 1;19(5):2883. doi: 10.3390/ijerph19052883 (PMC8910314; doi:10.3390/ijerph19052883)

Table S1. Items of the English Version of the General Dietary Behavior Inventory (GDBI-E) and Calculation of the GDBI-E score

Item code	Behavior A	Scoring					Behavior B
db1	I eat different foods every day.	5	4	3	2	1	I eat the same foods every day.
db2	My meals always include animal products (e.g., meat, fish, eggs, dairy products such as yogurt, cream, cheese).	5	4	3	2	1	My meals do not include animal products (e.g., meat, fish, eggs, dairy products such as yogurt, cream, cheese).
db3	My meals always include plant-based products (e.g., vegetables, grain products, tofu products).	5	4	3	2	1	My meals do not include plant-based products (e.g., vegetables, grain products, tofu products).
db4	I eat at least 2 servings of fruit daily.	5	4	3	2	1	I never eat fruit.
db5	I eat at least 3 servings of vegetables daily.	5	4	3	2	1	I never eat vegetables.
db6	I eat whole grain products (e.g., whole grain pasta, whole grain bread) instead of white flour products (e.g., "conventional" pasta, white bread/grain bread) every day.	5	4	3	2	1	I eat white flour products (e.g., "conventional" pasta, white bread/grain bread) instead of whole grain products (e.g., whole grain pasta, whole grain bread) every day.
db7	I always consume vegetable fats (e.g., margarine and oils based on canola, olive, sunflower) instead of animal fats (butter, lard, etc.).	5	4	3	2	1	I always consume animal fats (butter, lard, etc.) instead of vegetable fats (e.g., margarine and oils based on olives, canola, sunflowers).
db8	I do not eat sweets (e.g., chocolate, cookies, pastries).	5	4	3	2	1	I eat sweets (e.g., chocolate, cookies, pastries) every day.
db9	I do not eat fast food and convenience foods (e.g., frozen pizza, microwave ready-to-eat meals).	5	4	3	2	1	I eat fast food and convenience foods (e.g., frozen pizza, microwave ready-to-eat meals) daily.
db10	I do not drink sugary/sweetened beverages (e.g., juices, soda, sweetened coffee/tea).	5	4	3	2	1	I drink sugary/sweetened beverages (e.g., juices, lemonade, sweetened coffee/tea) daily.
db11	I consume at least 1.5 liters of fluid daily (excluding alcoholic beverages or beverages containing sugar/sweeteners).	5	4	3	2	1	I consume less than 1.5 liters of fluid (excluding alcoholic beverages or beverages containing sugar/sweeteners) daily.
db12(r)	When preparing meals, it is important to me that they are prepared quickly (e.g., quick frying, quick/spicy sautéing).	1	2	3	4	5	When preparing meals, it is important to me that they are prepared slowly (e.g., slow cooking/frying with medium heat).
db13	When seasoning my meals, I make sure to use as little salt as possible.	5	4	3	2	1	I like to use a lot of salt when seasoning my food.
db14(r)	I drink alcohol regularly (daily or even every weekend).	1	2	3	4	5	I don't drink alcohol or drink it very rarely (only on special occasions).
db15	I prefer to eat in peace without being distracted by anything.	5	4	3	2	1	While eating, I like to occupy myself with other topics (e.g., watching TV, thinking about work, reading).
db16	When taking my meals, I like to take my time.	5	4	3	2	1	I like to take my meals quickly.

GDBI-E score is calculated by adding up the scores given above for the items specified. For calculation of the GDBI-E score it is necessary that all items have been filled in and there are no missing values.

Figure S1. Stratified boxplot of GDBI-E score per general diet

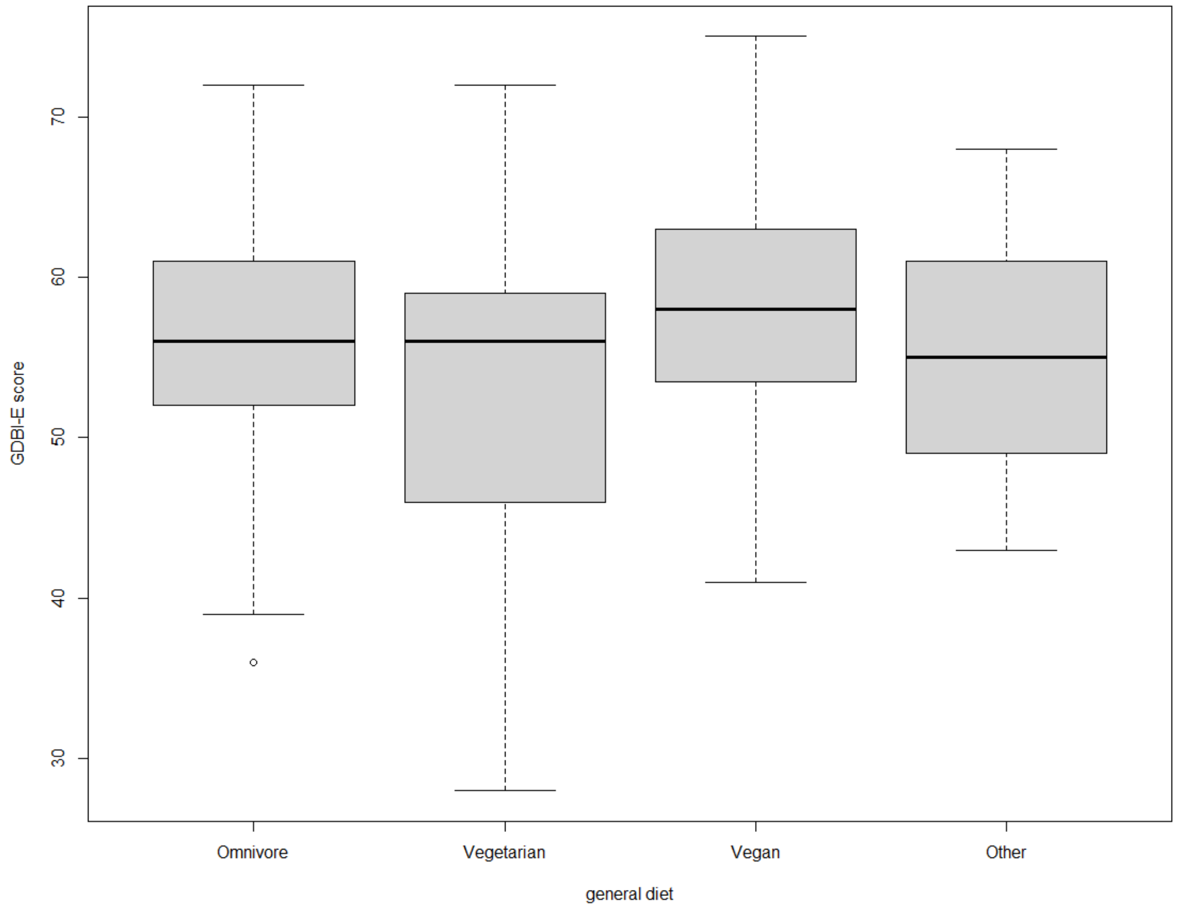

Supplement: Supplementary file 1 [file ijerph-19-02883-s001.zip › ijerph-1571563-supplementary.pdf]
